# Advancing the climate change and health nexus: The 2024 Agenda

**DOI:** 10.1371/journal.pgph.0003008

**Published:** 2024-03-28

**Authors:** Vanessa Kerry, Sadath Sayeed

**Affiliations:** 1 Seed Global Health: Boston, Massachusetts, United States of America; Lilongwe, Malawi; Freetown, Sierra Leone; Lusaka, Zambia; Kampala, Uganda; 2 Department of Global Health and Social Medicine, Harvard Medical School, Boston, Massachusetts, United States of America; 3 Division of Pulmonary and Critical Care, Department of Medicine, Massachusetts General Hospital, Boston, Massachusetts, United States of America; 4 Department of Pediatrics, David Geffen School of Medicine, University of California–Los Angeles, Los Angeles, California, United States of America; 5 Global Health Program, David Geffen School of Medicine, University of California–Los Angeles, Los Angeles, California, United States of America; St Luke’s Medical Center College of Medicine, PHILIPPINES

## Background

Climate change poses the most significant threat to human health in the 21st century, affecting nearly half of the world’s population. The negative impacts on health are already evident worldwide, with approximately 3.3 to 3.6 billion people at risk [1]. In November 2023, a new modeling study suggested over 8 million people a year die from outdoor air pollution; it also attributed a jarring 5 million deaths directly to fossil fuel use [2]. Over 60,000 deaths were attributed to extreme heat in Europe in the summer of 2022 [3]. Climate change is driving increases in malaria and other vector borne diseases [1], worsening mental health [4], burdens of noncommunicable diseases and endangering maternal and child health [5].

In addition to direct impacts of climate change on our human and planetary health, we are increasingly threatened by the economic and societal pressures it foments. Drought and excessive rainfalls are changing habitats, accelerating food insecurity and the risk of malnutrition. Basic sanitation and access to clean water remains under siege. The deleterious impacts of climate change are driving migration and insecurity, exacerbating pre-existing socio-economic inequities, and contributing to cycles of poverty and instability in many countries, undermining long-term economic growth and our progress towards the Sustainable Development Goals [6].

The climate crisis is a *health and well-being* crisis.

## Raising the Alarm

COP28 was a watershed moment for international climate negotiations punctuated by a year of extreme weather catastrophes from wildfires to extreme heat, to unprecedented rainfall and flooding, to drought. It included the first ever Day of Health, recognizing the inconvertible intersection of climate change and our human and planetary health. The day was a key signal to the global health community regarding the dangers of climate change and included funding announcements that have the potential to massively influence our collective response to climate and health priorities.

The Day of Health was highlighted by the first ever Ministerial Session on Climate Change and Health and welcomed over 110 Ministries of Health to join the proceedings. The Ministerial recognized the Climate Change and Health Declaration, a consensus document formally endorsed by over 140 countries that identified the health threats from climate change and the required responses. Importantly, a number of countries wrote specific addendums calling for stronger action than in the Declaration itself (See Box 1 for examples).

**Box 1. Example country specific addendums to COP28 Ministerial Declaration on climate and health**

Uganda:

“Despite strong national plans and ambition, Uganda’s health system remains under-resourced and under-staffed to meet the challenges posed by climate change. We know strengthening our health system including increasing the health workforce to provide high-quality health services will allow us to be better prepared to respond to the impacts of climate change. Further this health-centred response to climate change will have far-reaching effects across multiple sectors that will impact our population. These investments will require increased support from the global community to help us adapt to the changes impacting us daily. Insufficient financing is the main stumbling block to fully implementing national health and climate change plans. This climate-health financing gap threatens the lives of millions, leaves health systems without critical support, and deepens global health inequities. We need to overhaul the current financing systems. Debt is crippling many countries around the world, reinforcing cycles of poverty and ill-health. We need to help create the fiscal space needed for governments to make the necessary investments in health.”

Zambia:

“We call for all countries to adopt a just transition from fossil fuel use and to reduce greenhouse gas emissions urgently. Zambia’s, like many other less resourced countries’ health systems, are highly vulnerable to climate change and our populations bear disproportionate health burdens from these fossil fuel emissions.

A committed leader to reducing greenhouse gases and champion to transition to affordable, reliable, and accessible clean energy, Zambia took a bold step to end fossil fuel subsidies in 2021, directing these funds to social protections for our people instead. The financial burden of this has been felt by all Zambians. Yet, poorer countries like Zambia should not have to bear the full burden of such reform. As we call on all countries to follow our lead and commit to ending fossil fuel subsidies, we also urge richer nations to support less-resourced countries to achieve this–to underwrite these reforms–which will benefit our earth and promote the health and well-being of all.”

## Accelerated action

Now is the time for aggressive action. At the close of COP28, parties eked out language that called for “a transitioning away from fossil fuels.” The wording was a first; fossil fuels were finally codified publicly as a driver of climate change. However, the language remains vague and nuanced. There is no option but to phase out fossil fuel use. We face an unsustainable global temperature rise of 2.4 to 2.6 degrees Celsius by the end of the century [7]. The science and technology are simply not yet in place to abate greenhouse gases from fossil fuel use significantly enough to hold the world to 1.5 degrees [8]. Urgent ‐ and concrete plans ‐ for mitigation and making a full shift to renewable energy are required to avoid overwhelmingly more negative human health consequences and prevent the loss of key natural resources and ecosystems that are critical to both human and non-human species health [9].

## Resilient health systems

As we look to answer the challenges at the climate change and health nexus, the global health community has an opportunity to make smarter, across-the-board health system strengthening investments. COVID revealed our vulnerabilities to large-scale shocks. During the pandemic, we saw years of progress reversed and a near collapse of health systems globally as over 90 percent of countries saw disruptions in health services [10], leading to increased deaths from preventable causes, as well as trillions in GDP losses [11] and a surge in poverty [12]. Wealthy nations in particular, with a relative abundance of resources, failed to meet the moment.

To effectively and meaningfully meet the health crises being wrought by climate change, we need to examine how we can *comprehensively* build resilient health systems, how to fund them most impactfully and how to align behind strong national visions. Governments must be supported around holistic plans that incorporate the threat of climate change and its increasing pressure on fragile health systems into their strategies. We must end our far-too-long favored and easy tendency to think along disease-specific lines in scaling programs and interventions. For too long these and other vertical approaches that reinforce disturbing power asymmetries have chopped up health delivery systems. They have complicated and frustrated the development and implantation of national plans for countries as they instead have been forced to cobble together disconnected responses that cater to the specific interests of outsized influential external funders. They have created administrative headaches, redundancies, and costs that waste precious bandwidth, time, and focus.

## Financing

We need more, flexible, accessible, and accelerated financing from wealthier governments and philanthropy to unlock our ability to address the negative health impacts due to climate change. Much attention at COP was on the passage of the long-awaited Loss and Damage Fund (L&D). However, the L&D is a not a health-specific fund and saw only $450 million in pledges ‐ well below the $387 billion [13] estimated to be needed.

The Health Day was highlighted by an announcement of $1B in funding dedicated to the climate and health nexus. The Global Fund for AIDS, Tuberculosis, and Malaria led with $512 million, and both Wellcome Trust and Rockefeller Foundation pledged around $100 million each. However, over half of this funding has already been earmarked for expenditure or is dedicated to vertical siloed projects [14]. In other words, much of it is not new. There is a risk now that these funds will be perceived as readily available for novel strategic initiatives, when they are likely not. And with this seemingly momentous announcement, there is a risk of complacency to raise new sources of funding.

At the crux of any honest climate and health discussion must be the acknowledgment that while we already know what to do, those of us with the power to seriously rethink our approach lack the will to do it. We must now choose to do it. A health centered climate response which focuses on building resilient, capable of shock-withstanding, *entire* health systems will save millions of lives. It will also strengthen every country’s economy, and help nurture a positive cycle of human well-being and productivity. The economic return of every dollar invested in health is at least $4 [15]. Healthy people grow economies, create jobs, and foster social inclusion and gender equity.

We must correct course immediately if we have any hope of avoiding the deadliest impacts of climate change in the years ahead.
